# 219. Safety and Immunogenicity of BNT166a, an Investigational Quadrivalent mRNA Mpox Vaccine

**DOI:** 10.1093/ofid/ofaf695.077

**Published:** 2026-01-11

**Authors:** Leela Davies, Frank Bähner, Saul N Faust, Olga Blokhina, Donald Brandon, William B Smith, Vengalil Krishna K Chatterjee, Hana Hassanin, Tara M Babu, Adam Zuiani, Tim Müller, Asaf Poran, Claire Brittain, Jay W Hooper, Eric Mucker, Robbert G van der Most, Pedro Alonso, Federico Mensa

**Affiliations:** BioNTech US Inc, Cambridge, Massachusetts; BioNTech SE, Mainz, Hamburg, Germany; University of Southampton and University Hospital Southampton NHS Foundation Trust , Southampton, England, United Kingdom; BioNTech SE, Mainz, Hamburg, Germany; California Research Foundation, San Diego, CA; Alliance for Multispecialty Research, Knoxville, Tennessee; NIHR Cambridge Clinical Research Facility, Cambridge, England, United Kingdom; University of Surrey, Guildford, England, United Kingdom; University of Washington, Seattle, Washington; BioNTech US Inc, Cambridge, Massachusetts; Staburo GmbH, Munich, Bayern, Germany; BioNTech US Inc, Cambridge, Massachusetts; BioNTech UK Ltd, London, England, United Kingdom; United States Army Medical Research Institute of Infectious Diseases, Fort Detrick, Maryland; United States Army Medical Research Institute of Infectious Diseases, Fort Detrick, Maryland; BioNTech SE, Mainz, Hamburg, Germany; BioNTech SE, Mainz, Hamburg, Germany; BioNTech, Mainz, Rheinland-Pfalz, Germany

## Abstract

**Background:**

Mpox remains a public health emergency of international concern. The emergence of new variants emphasizes the need for novel, durable, and scalable vaccines against monkeypox virus (MPXV). BNT166a is a lipid nanoparticle-encapsulated mRNA vaccine candidate encoding MPXV antigens A35, B6, M1, and H3. It has demonstrated preclinical immunogenicity and protective efficacy against orthopoxviruses including MPXV clades I and II and vaccinia virus (VACV) in murine and non-human primate models.

**Methods:**

In a first-in-human Phase 1 clinical trial (NCT05988203), VACV-naïve participants were assigned to receive two intramuscular injections of BNT166a at doses of 10, 30, or 60 µg administered four weeks apart. VACV-experienced participants were assigned to receive two vaccinations (Vx) of 30 µg BNT166a on the same schedule. The primary endpoint was safety, with exploratory endpoints assessing immunogenicity through one-year post-Vx 2.

**Results:**

Forty-eight VACV-naïve participants (median age 35, 50% female) and 16 VACV-experienced participants (median age 58, 69% female) were enrolled and received at least one Vx, with 92% completing the two-Vx schedule. Local and systemic reactogenicity events were mostly mild-to-moderate and more frequently observed post-Vx 2 than post-Vx 1. The most common were injection-site pain, fatigue, headache, muscle pain, and chills. BNT166a-related unsolicited adverse events (AEs) were mostly mild-to-moderate. No related serious AEs or AEs of special interest were observed through 201 days post-Vx 2. BNT166a induced binding antibodies against all four mRNA-encoded antigens in all participants, both VACV-naïve and -experienced, which persisted through six months post-Vx 2. Neutralizing antibodies to the mature virion form of MPXV clades I and IIb and VACV, and the extracellular virion form of VACV, were elicited in VACV-naïve groups and boosted in VACV-experienced participants by two weeks post-Vx 2. Immunogenicity data through 12 months post-Vx 2 will be presented at ID Week.

**Conclusion:**

BNT166a is well-tolerated and induces multiantigen-directed antibodies with cross-MPXV clade and cross-orthopoxvirus neutralization activity in VACV-naïve and -experienced participants, supporting its advancement to Phase 2 trials.

**Disclosures:**

All Authors: No reported disclosures

